# Radical Cure of Umbilical Hernia in a Colt*From Wochenschrift für Thierheilkunde. No. 24, 1884.

**Published:** 1884-10

**Authors:** Eugene Frohner

**Affiliations:** Stuttgart


### CASE DEPARTMENT.

### I.—RADICAL CURE OF UMBILICAL HERNIA IN A COLT.*

BY PROF. EUGENE FROHNER, STUTTGART.

A year-old colt was brought to the hospital of the Veterinary School at Stuttgart on the 9th of February, 1884. When about 3 weeks old, a swelling was observed in the umbilical region being about the size of a pigeon's egg, which gradually increased in size. It was attributed to the sudden getting up of the colt from having been startled. Some months previous to my seeing him, some one had endeavored to cure the trouble by running a sharp nail through a portion of the sack with a ligature over it.

At the time of observation the swelling was about the size of a man's fist, and somewhat warmer than its surroundings. Its contents could be easily pushed upward, and one could feel an oval opening some 8 to 10 centimetres long, its edge being indented. The anterior end was rounded off, the inferior or posterior being more wedge-shaped.

The indications for a radical cure were by no means favorable, the patient being in poor condition, abdomen very pendulent, visible mucosae icterical, temperature, 39.2°C., bronchial catarrh, and some dull spots in lungs on percussion—aside from this the animal was weak and very apathetic.

On this account the operation was put off until the 14th, and attention was given to diet and stimulation. On that date we operated, chloroform not being given or even required on account of the general condition of the patient.

*Operation.*—The hair was first clipped off from the skin on and about the hernia, the entire locality being carefully washed and cleaned with an aqueous solution of Sublimate. A spray of the same material was diligently applied during the operation. On opening the sack a large amount of yellowish serum escaped. Reposition of the contents was easily performed. The edges of the canal were then freshened and brought together with a sufficient number of deep and superficial strong silk sutures.

In 13 days the animal was turned over to the owner completely healed, union having occurred directly of the edges of the canal.

The only complication was the most unexpected one of Testo-vaginitis, probably due to a moderate peritonitis which extended in this direction. No serious consequences resulted, however.

### II.—A CASE OF ENTERITIS.

BY ALVAH C. VAN SYCKLE, M.D.

In order to give a careful history of this case, I should state that there has been prevailing in this locality an infectious disease resembling distemper.

It comes under a nomenclature of the scarlet fever type in childhood. Most of the horses begin with malaise, followed in 48 hours with a cough,

*From Wochenschrift für Thierheilkunde. *No.* 24, 1884.

